# Comparing the efficacy of a multi-dimensional breast cancer rehabilitation programme versus a home-based exercise programme during adjuvant cancer treatment

**DOI:** 10.1186/s12885-024-12080-5

**Published:** 2024-03-21

**Authors:** Gobinathan Chandran, Ning Tang, Ednajoy Lay Poh Ngo, Serene Huang, Shuk In Tong, Jie Xin Ong, Effie Chew

**Affiliations:** 1 Department of Medicine, Division of Rehabilitation Medicine, National University Hospital, NUHS Tower Block, Level 10, 1E Kent Ridge Road, Singapore, 119228 Singapore; 2 Yong Loo Lin School of Medicine, National University of Singapore, 10 Medical Drive, Singapore, 117597 Singapore; 3 Division of Oncology Nursing, National University Cancer Institute, 5 Lower Kent Ridge Road, Singapore, 119074 Singapore; 4 Department of Rehabilitation, National University Hospital, 5 Lower Kent Ridge Road, Singapore, 119074 Singapore

**Keywords:** Breast cancer, Multi-dimensional rehabilitation programme, Supervised exercise, Home-based exercise

## Abstract

**Background:**

Breast cancer is the most common female malignancy worldwide and a major cause of morbidity and mortality. Exercise during adjuvant treatment improves function and relieves symptoms in breast cancer survivors. However, it is unclear if an unsupervised exercise programme may be as effective as a supervised multimodal group. We investigated the feasibility and efficacy of a centre-based multidimensional rehabilitation (MDR) programme for breast cancer survivors undergoing adjuvant treatment and compared it to an unsupervised home-based exercise (HE) programme.

**Methods:**

Participants were self-allocated to either MDR or HE group. MDR participants underwent 24 supervised exercise classes and 10 education classes over 12 weeks. HE participants were instructed on a home exercise regime. Outcome measures, including the 6-min walk test (6MWT) and Frenchay Activities Index (FAI), FACT-Cognitive Function scale, and European Organization for Research and Treatment of Cancer Quality of Life Questionnaire-Core 30, were conducted at baseline (W0), post-intervention (W12) and 6-months post-intervention (M6). Variance between time points and the 2 groups were analysed using a linear mixed model (unstructured covariance matrix) and adjusted with Bonferroni.

**Result:**

Twenty-five participants attended at least half of the MDR interventions, while 21 completed the HE interventions. The former showed significant improvement in 6MWT, from 406.88 m (W0) to 443.34 m (W12) to 452.81 m (M6), while the improvement in the HE group was not significant (407.67 m (W0) to 433.14 m (W12) to 430.96 m (M6)). Both groups showed a significant improvement in FAI, with earlier significant improvement noted at W12 in the MDR group (22.71 (W0) to 27.65 (W12) to 28.81 (M6)) compared to the HE group (23.16 (W0) to 26.47 (W12) to 29.85 (M6)). Dropout rate was 16% in the MDR group and 34% in HE group. Overall satisfaction with the MDR programme was high.

**Conclusion:**

Both MDR and HE programmes were feasible. MDR was superior in improving endurance and earlier return to instrumental activities for those who completed at least half of the sessions. Future studies could explore use of technology to improve adherence to exercise.

**Trial registration:**

The study was registered with ClinicalTrial.gov on 01/04/2022 with the registration number NCT05306808.

## Background

Breast cancer is the most prevalent cancer among women in Singapore [1]. Breast cancer and its treatment commonly result in fatigue, depression, impairment of cardiorespiratory function and muscle weakness [2]. Moderate to severe fatigue is reported in 30–60% of patients during cancer treatment [3]. An average reduction of strength of 25% in lower extremities and 16% in the upper extremities has been reported in breast cancer patients during treatment [4].

Physical activity and exercise interventions at all stages post-diagnosis have been shown to improve aerobic capacity, strength, quality of life, body image, prevent and manage fatigue, pain, depression, weight gain, and survival, including sarcopenia-related mortality [5–9]. Besides symptom control, physical activity also affects the prognostic outcomes and rate of recurrence in breast cancer [10]. Various models of cancer rehabilitation programmes have been shown to be cost-effective [11] and cancer rehabilitation has been included in survivorship guidelines and recommendations around the world [12]. The focus for exercise during adjuvant treatment is the attenuation of adverse effects of treatment including fatigue and cognitive impairment, to improve tolerability of adjuvant treatment, and to improve physical fitness and strength [13, 14].

Despite the benefits, most breast cancer survivors do not adhere to exercise recommendations and rehabilitation prescriptions [15]. In fact, breast cancer survivors have been reported to reduce their physical activity levels after diagnosis by an average of 2 h a week, or deceased by 11%. Even more reduction in physical activity levels (50%) was observed in those who underwent surgery, radiation and chemotherapy [16]. Education is an integral part of cancer rehabilitation and improves self-management skills, empowers cancer survivors and improves self-efficacy for managing symptoms and results in less distress, better psychosocial adjustment and satisfaction among cancer survivors [17, 18].

In Singapore, while cancer surveillance rates are high [19], less value has been placed on function and exercise, with 46.1% of survivors reporting to have received exercise guidance from healthcare professionals post-diagnosis, and 52% reporting side effects of treatment as a barrier to exercise [20]. Less than half of those surveyed reported reduction of adverse effects as an impetus to exercise [20]. Similarly, another survey reported 54% of those presenting with functional impairments were not willing to undergo rehabilitation [21], reflecting a need for better education. While supervised centre-based programmes have advantages in enhancing adherence to exercise, home-based exercise programmes are convenient and effective as well [22].

Therefore, we undertook a programme addressing education and therapeutic exercise for breast cancer survivors while on adjuvant cancer treatment, with the aim to evaluate the feasibility of the multi-dimensional rehabilitation programme and to compare its efficacy with a home exercise programme.

## Methods

### Participants

Breast cancer survivors attending the outpatient oncology clinic at the National University Cancer Institute Singapore were screened for eligibility to participate in this study. Women 21 to 80 years old receiving active treatment (chemotherapy, radiation therapy or targeted therapy) for breast cancer, and being able to walk independently without aid, were offered participation. Patients were excluded if they were pregnant, had uncontrolled medical conditions or conditions limiting active participation in group exercise (e.g. those with fracture risk, neuromusculoskeletal conditions requiring individualised rehabilitation); or were already participating in regular moderate to high intensity physical activity. The study was registered with ClinicalTrial.gov on 01/04/2022 with the registration number NCT05306808.

### Study design

This trial was reported as per the SPIRIT 2013 Guidelines. This was a non-randomised assessor-blinded controlled trial. Participants were given the option to be either in the multidimensional rehabilitation (MDR) group or the home exercise (HE) group so as to improve recruitment and limit dropouts.

At the beginning of the programme, both groups underwent an occupational therapy session to assess their functional limitations and coping strategies in managing their symptoms. Participants in the MDR group undertook 24 sessions of hospital-based exercise class and 10 sessions of education class over a duration of 12 weeks. The supervised 1-h group exercise classes were conducted twice a week by a physiotherapist with an assistant, with a maximum of 8 participants in each class. Participants who were unable to attend a class for any reason were advised to perform exercises at home. Home exercise prescription was provided by physiotherapist for all participants, targeting moderate intensity aerobic and strength training (Table 1). Education classes were conducted once a week for participants in the MDR group, by a multidisciplinary team including physiotherapists, occupational therapists, advanced practice breast care nurses, dietician and medical social worker. Topics included physical activity, managing fatigue, lymphoedema and peripheral neuropathy, cognitive impairment, arm care after surgery, optimising nutrition, managing stress, managing relationships and return to work. Participants in the HE group had a single physiotherapy session where they were instructed on a home exercise programme which was undertaken without supervision for 3 months. Both groups were asked to keep a log of their home exercise performance. At the end of 12 weeks, both groups attended a 2-h survivorship transitional class conducted by the advanced practice nurse, with topics covering cancer surveillance and follow-up, addressing fear of recurrence, screening for colorectal and cervical cancers, community reintegration and resources.

**Table 1:** Exercise prescription for the MDR and HE group

	**MDR Group**	**HE Group**
Frequency (number of sessions per week)	2 sessions of supervised exercise + 1 session of unsupervised exercise	5 sessions of unsupervised exercise
Duration per session	Supervised exercise: 60 min Unsupervised exercise: 30 min of aerobic exercise or strengthening exercise	Unsupervised exercise: 30 min of aerobic exercise or strengthening exercise
Exercise duration per week	150 min	150 min
Total duration of the exercise programme	12 weeks	12 weeks
	**Supervised Exercise**	**Unsupervised Exercise**
**Aerobic Exercise**		
Example	Arm Ergo, Treadmill, Elliptical, Cycling, Rower, Step-ups	Walking, Cycling/leg pedal, Marching on the spot, Step-ups^a^
Duration per session	30 min	30 min
Intensity RPE, 6–20 Borg Scale	11–13	11–13
Eliciting progressing overload Recommendations	Increase speed, resistance, or incline to maintain 40% to 60% (moderate intensity) of heart rate reserve or RPE	Increase speed or duration to maintain 40 to 60% (moderate intensity) of heart rate reserve or RPE
**Strengthening Exercise**		
Exercise tools	Resistance bands, dumbbells, machine-weights and calisthenics	Resistance bands and calisthenics
Example of upper body strengthening	Scapular retractions, Shoulder external rotation, Shoulder internal rotation, Shoulder press, Latissimus dorsi pulldown, Bicep Curls, Triceps, Pectorals with chest press	Shoulder internal rotation, Shoulder external rotation, Shoulder press, Shoulder abduction, Chest expansion, scapular retraction, Latissimus Dorsi pull, Triceps, Biceps
Example of lower body strengthening	Hip extension, Wall Squat, Knee extensions, Step-ups, Hip abduction, Heel raises	Hip flexion, Hip extension, Hip abduction, Knee extension, Wall squats, Bridging, Heel raises
Duration per session	30 min	30 min
Intensity RPE, 6–20 Borg Scale	11–13	11–13
Repetitions in reserve	10–15 repetitions × 2–3 sets	10–15 repetitions × 2–3 sets
Exercise recommendations	3 to 4 major UL muscle groups 3 to 4 major LL muscle groups	3 to 4 major UL muscle groups 3 to 4 major LL muscle groups
Eliciting progressive overload	Increase reps or sets, increase resistance or weights	Increase reps or sets, increase resistance or weights

*Abbreviations*: *MDR* Multidimensional rehabilitation, *HE* Home-exercise, *RPE* Rate of perceived exertion, *UL* Upper limb, *LL* Lower limb

^a^This is not an exhaustive list; if a participant wanted to engage in other aerobic exercises, it could be included in the exercise prescription if it was deemed safe and appropriate to the participant’s goal

### Outcome measures

Outcome measures were collected at baseline (W0), post-intervention (W12) and 6-months post-intervention (M6). Post-intervention data was collected regardless of the number of sessions participants missed. The 6-min walk test (6MWT) [23] was used to assess changes in aerobic capacity. Perceived changes in the domains of cognitive function and fatigue were specifically investigated using the Functional Assessment of Cancer Therapy –Cognitive Function (FACT–Cog) [24] and the FACT-fatigue [25] scales respectively. The FACT–Cog has 4 subscale domains: CogPCI (Cognitive function- perceived cognitive impairment), CogOth (Cognitive function- comments from others), CogPCA (Cognitive function- perceived cognitive abilities), and CogQOL (Cognitive function- impact of perceived cognitive impairments on quality of life). The Frenchay Activities Index (FAI) [26] was used to assess participation in instrumental activities of daily living. European Organization for Research and Treatment of Cancer Quality of Life Questionnaire-Core 30 (EORTC QLQ-C30) [27] was used to measure quality of life in patients with cancer. Raw data was re-calculated according to the manual of the EORTC QLQ-C30 to obtain the final score for each of the dimensions/scales.

Participants in the MDR group were given a feedback questionnaire at the end of each education class, where they rated whether (1) the objective of the programme was met; (2) the content covered during the session was sufficient; (3) the duration of the programme was sufficient; (4) the questions were easy to understand; and (5) the programme was useful, (6) overall satisfaction with the programme and (7) whether the class size was appropriate for the education sessions.

### Statistical analysis

All statistical analyses were undertaken using the SPSS version 23 software. Student t-test was used to analyse the baseline differences between two groups. Variance between time points and the 2 groups (the MDR and HE) were analysed using a linear mixed model (unstructured covariance matrix) and adjusted with Bonferroni. Statistical significance was calibrated at *P* < 0.05.

## Result

**Fig. 1:**
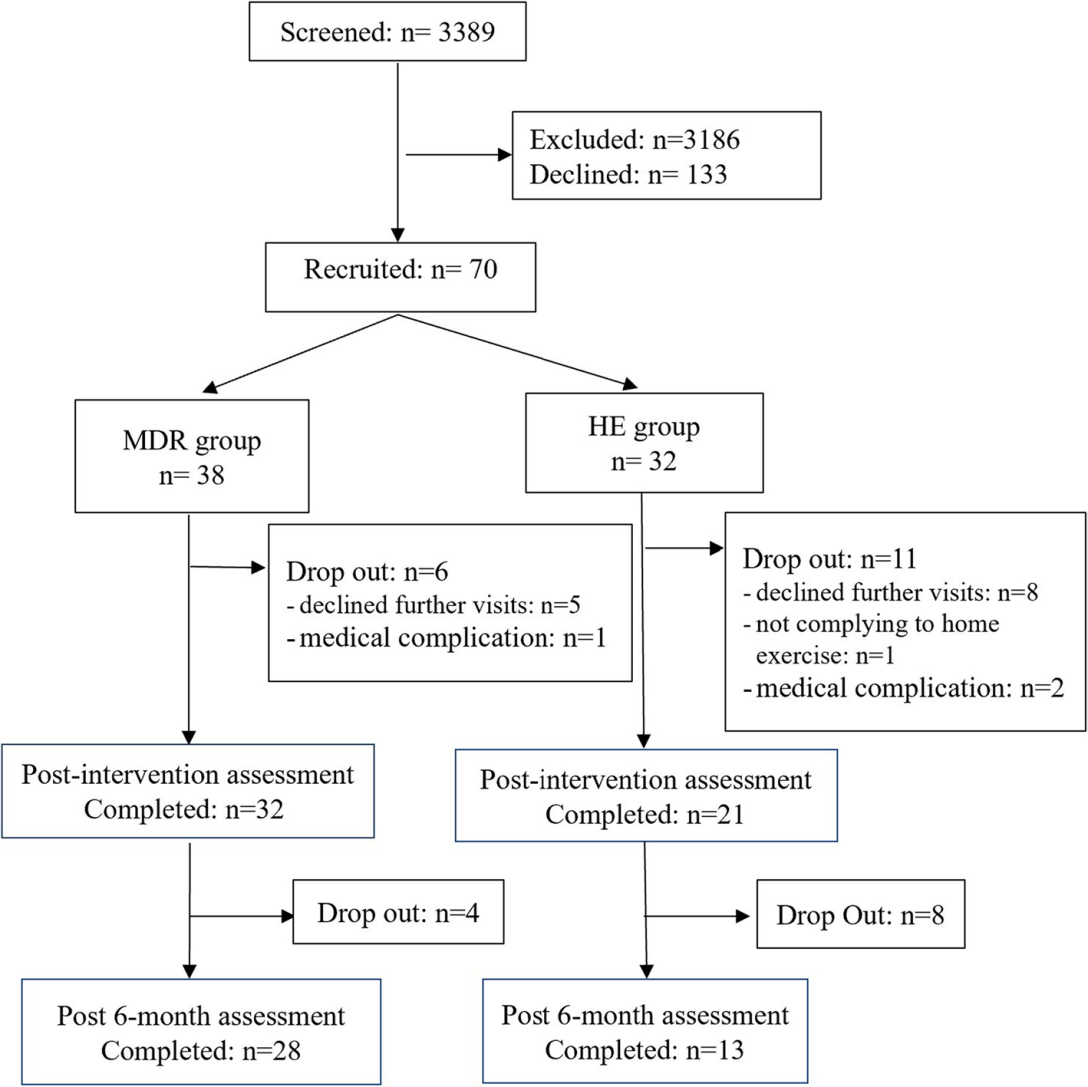
CONSORT diagram of participant recruitment

Of 3389 breast cancer survivors screened, 203 qualified for participation and 70 were recruited. 53 completed the study interventions and 41 completed M6 assessment (Fig. 1). There were no significant differences between groups at baseline in terms of age, cancer stage and type of treatment undertaken (Table 2). Body mass index (BMI) was higher in the MDR group than the HE group.

**Table 2:** Demographic information of participants

		HE (*n* = 21)	MDR (*n* = 32)	Difference between groups
Age (Mean (SD)		48.62 ± 11.78	51.78 ± 9.56	*p* > 0.05
BMI (Mean (SD)		23.29 ± 3.11	26.36 ± 6.33	*p* = 0.045
Cancer stage (n)	IA	4	4	*p* > 0.05
	IB	1	1	
	IIA	10	10	
	IIB	4	5	
	IIIA	1	9	
	IIIB	1	-	
	IV	-	1	
	Unknown	-	1	
Treatment (n)	Chemotherapy	10	18	*p* > 0.05
	Targeted therapy	2	5	
	Chemotherapy &Targeted therapy	9	8	
	Surgery	12	23	

In the MDR group, 78% attended at least 50% of exercise sessions, the mean exercise attendance rate was 67.6% and the median was 72.9%. The most common reasons for absence were feeling unwell, or being hospitalized. In the HE group, 65.6% completed study interventions.

### Aerobic capacity

**Fig. 2:**
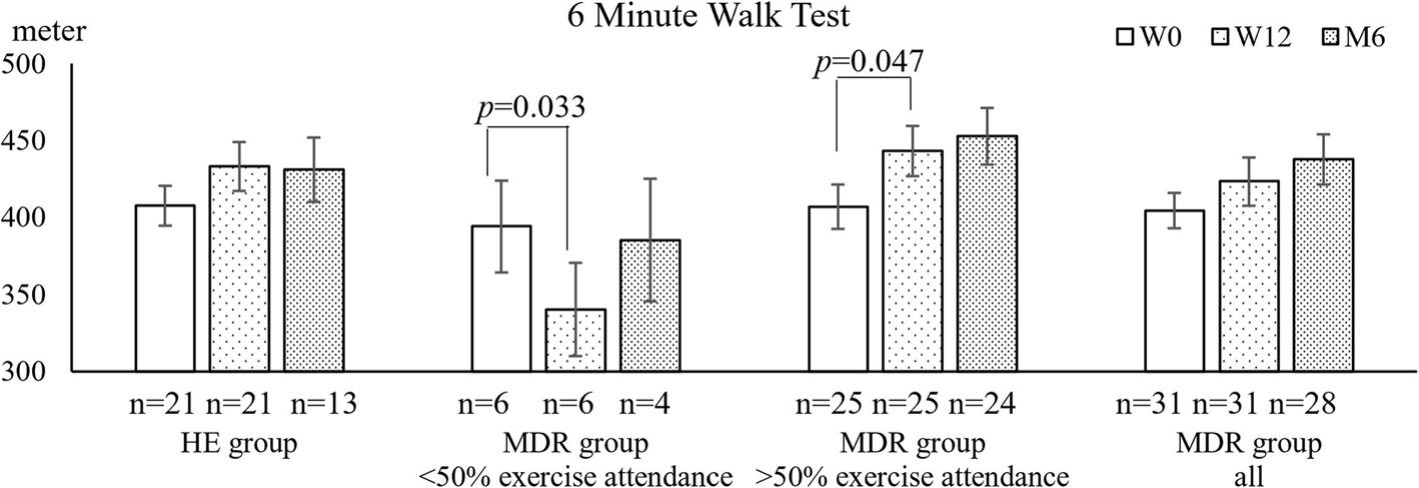
6MWT outcomes at W0, W12 and M6. Data shows Mean and SEM

There was a trend towards improved aerobic capacity as measured by 6MWT over time in both the MDR and HE groups, which was not statistically significant (Fig. 2). However, when considering only those who attended at least 50% of the exercise sessions, significant improvement in aerobic capacity was seen over time in the MDR group (*F* = 4.307, *p* = 0.025). Post-hoc analysis showed improvement in 6MWT between W0 and W12 (*p* = 0.047), while those who attended < 50% of exercise sessions had a significant decrease in aerobic capacity at W12 (*p* = 0.033). The overall difference between “ < 50% attendance group” and “ ≥ 50% attendance group” is statistically significant (*F* = 4.405, *p* = 0.044). No difference was demonstrated between those who attended < 50% of exercise and the HE group.

### Instrumental activities of daily living

**Fig. 3:**
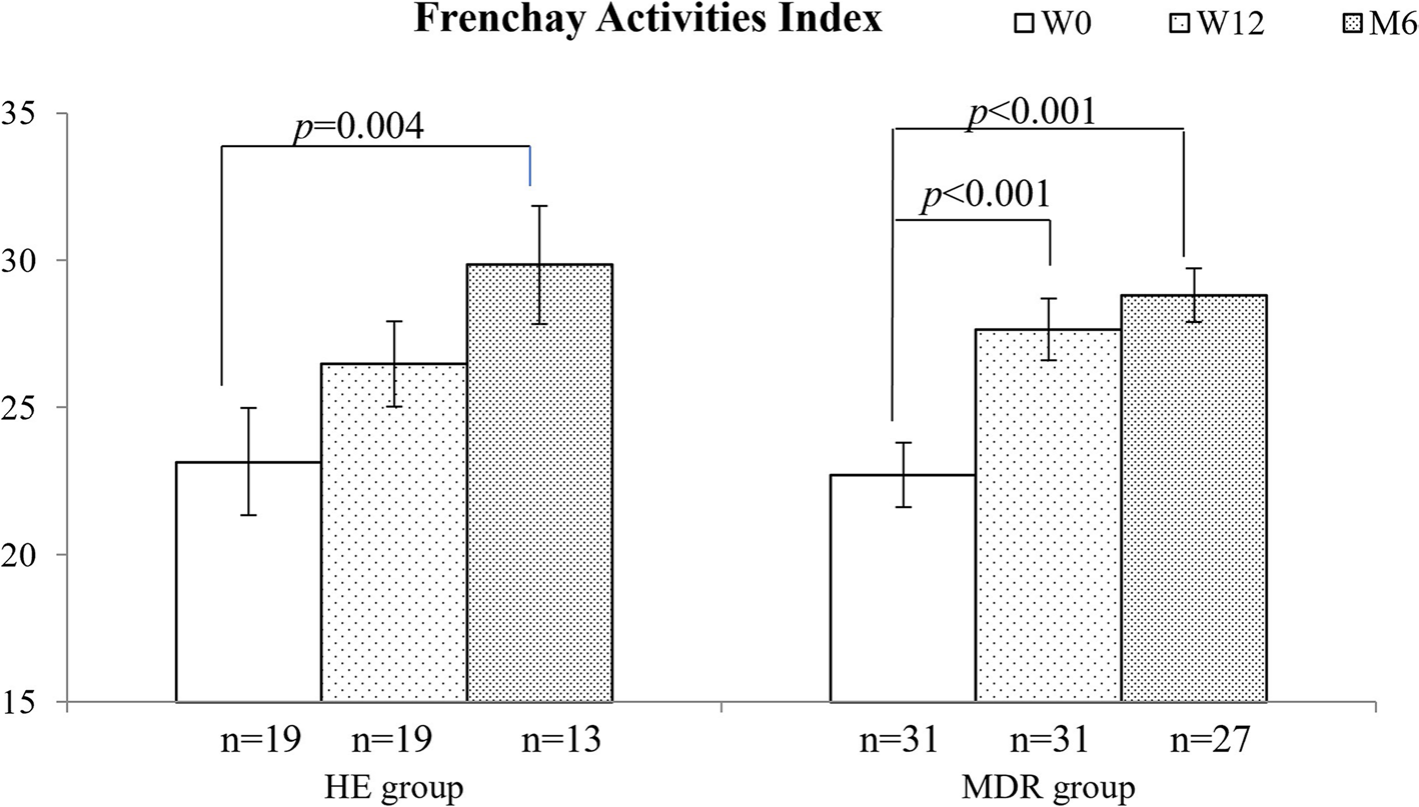
FAI outcomes at W0, W12 and M6. Data shows mean and SEM

Both groups showed significantly improved in instrumental activities of daily living (iADL) as measured by FAI (*F* = 7.941, *p* = 0.004 for the HE group; *F* = 19.110, *p* < 0.001 for the MDR group. Fig. 3). The MDR group had significant improvement between baseline and W12 and M6, whereas the HE group demonstrated significant improvement only between baseline and M6. Between group differences were not significant (*F* = 0.052, *p* = 0.820).

### Cognitive function and fatigue

Overall, 9 of 51 (17.6%) participants from both MDR and HE groups had perceived cognitive impairment at baseline compared to 12 of 51 (23.5%) at W12 (score of ≤ 54 on the FACT CogPCI domain). A significant decline in perceived cognitive ability (CogPCA) was observed in the MDR group. Bonferroni comparison showed significant decline between W0 and W12 (*p* = 0.010), and M6 (*p* = 0.012). There was no significant change in the HE group. No significant changes were found in other FACT-Cog domains and in FACT-fatigue between groups, or over the 3 time points for each group (Table 3).

**Table 3:** FACT outcomes at W0, W12 and M6

		W0	W12	M6	Within group difference
CogPCI	HE	62.35 ± 12.91 (*n* = 20)	62.10 ± 9.19 (*n* = 20)	66.23 ± 8.08 (*n* = 13)	not significant
	MDR	60.78 ± 12.48 (*n* = 31)	57.16 ± 12.45 (*n* = 31)	55.00 ± 13.85 (*n* = 28)	not significant
CogOth	HE	15.50 ± 0.83	15.30 ± 1.59	15.38 ± 0.96	not significant
	MDR	15.29 ± 1.42	14.84 ± 2.18	14.70 ± 2.05	not significant
CogPCA	HE	23.05 ± 3.73	21.05 ± 5.28	23.54 ± 3.48	*F* = 1.487, *p* = 0.253
	MDR	22.81 ± 4.53	20.19 ± 5.28^a^	18.38 ± 6.30^a^	*F* = 7.839, *p* = 0.002*
CogQOL	HE	14.60 ± 2.26	14.75 ± 2.45	14.31 ± 3.25	not significant
	MDR	14.06 ± 3.14	13.74 ± 3.53	13.00 ± 3.98	not significant
FACT-Fatigue	HE	40.35 ± 10.45	39.20 ± 11.41	44.31 ± 5.78	not significant
	MDR	36.53 ± 10.43	38.63 ± 10.94	39.36 ± 10.30	not significant

Data shows Mean ± SD

*Abbreviations*: *FACT* Functional Assessment of Cancer Therapy, *CogPCI* Cognitive function- Perceived Cognitive Impairment, *CogOth* Cognitive function- comments from others, *CogPCA* Cognitive function- perceived cognitive abilities, *CogQOL* Cognitive function- impact of perceived cognitive impairments on quality of life, *HE* home-based exercise; MDR: multidimensional rehabilitation programme

^*^Indicates *p* < 0.05

^a^Indicates statistically significant difference compared to W0

### Quality of life

Significant improvement in EORTC QLQ-C30 social functioning was noted in the HE group over time (*F* = 7.714, *p* = 0.004), between W0 to M6 (*p* = 0.009), and W12 to M6 (*p* = 0.006). The HE group reported less fatigue at M6 compared to W0 (*p* = 0.003), and W12 (*p* = 0.001). There were no significant differences in other EORTC QLQ-C30 functioning and symptom scales between the 2 groups, or over the 3 time points.

### Feedback on the education sessions

**Fig. 4:**
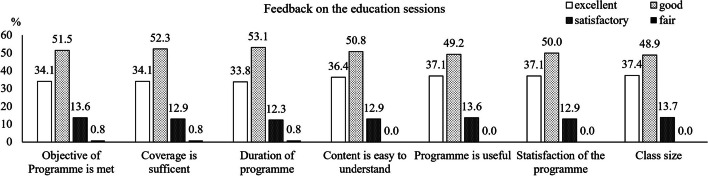
Feedback on the education sessions. Data shows the percentage of respond from poor, fair, satisfactory, good and excellent to each of the questions. No participants reported poor for the education sessions

More than 85% of participants reported a ranking of ≥ 4 (good and excellent) for the different components of the programme content (Fig. 4).

## Discussion

A 12-week multidimensional rehabilitation programme incorporating moderate intensity group exercise and education was effective in improving aerobic capacity in those who attended at least half of the exercise sessions (≥ 12 sessions over 12 weeks). Improvement in independence in iADL was noted earlier at W12 for the MDR group, as compared to only at M6 in the HE group, suggesting that the programme may facilitate earlier return to independent functioning in breast cancer survivors receiving active cancer treatment. No difference was reported in FACT-fatigue between groups and over time. The findings of less fatigue and better social functioning at 6 months in the HE group may be due to the lower proportion of advance stage cancer survivors and high number of dropouts in the HE group at 6 months. Other EORTC subscales did not demonstrate significant changes.

Our findings are consistent with previous reports of benefits of exercise during adjuvant treatment to improve aerobic capacity (SMD 0.42) and reduce fatigue (SMD -0.28) [14]. The benefits on health-related quality of life is equivocal [28]. The small effect we found in aerobic capacity is consistent with findings that breast cancer survivors undergoing adjuvant treatment had smaller improvements compared to those undergoing exercise after completing adjuvant treatment [29], consistent with the goal to alleviate symptoms and maintain function during treatment. Another study is also supportive that exercise during adjuvant therapy preserved physical function, which was evidenced by improved lower limb strength and self-reported physical function [30].

Our finding of baseline subjective cognitive impairment in 17.6–23.5% of our cohort is consistent with the prevalence of cancer-associated mild cognitive impairment reported in breast cancer survivors of 10–40% [31–33]. Memory and attention are the most commonly reported problems [31]. Our study did not show improvement in cognitive ability with exercise intervention. On the contrary, the MDR group reported worsening of perceived cognitive abilities after intervention, which may have been due to the cognitive burden from concurrent participation in the centre-based intervention sessions while on adjuvant treatment. Additionally, the educational sessions might have also increased the participants’ awareness in detecting cognitive changes. Studies of the effect of exercise on cognition in cancer patients have yielded inconsistent results. More studies are recommended to ascertain the benefits of exercise for cancer-related cognitive functioning which should include both self-reported and objective outcomes [28, 34].

Satisfaction with the education sessions conducted by the multidisciplinary team was high in the MDR group. Multiple studies have demonstrated the benefits of patient education in the management of pain [35], fatigue [36], and psychosocial and self-management skills [17] in cancer patients. Such education sessions aimed on self-management should be standard of care in cancer management [35].

Our study compared a supervised exercise programme with education, versus home exercise prescription with compliance monitoring and found both programmes feasible. We found greater adherence in the MDR group compared to HE, with a dropout rate of 16% in the MDR group compared to 34% in the HE group during the intervention phase, with no complications attributable to the interventions. A number of studies have demonstrated greater benefits of supervised exercises compared to home-based over unsupervised exercises [13, 37]. Dosage and intensity of exercise can be better monitored during supervised sessions, and a dose–response relationship has been demonstrated with supervised exercise, to improve cancer-related fatigue [8]. Other key benefits include better adherence, better tailoring of exercise prescription and targeting of functional deficits, and greater perceived efficacy of interventions [13, 38]. Better adherence is also associated with specific characteristics of the exercise prescribed, including type, intensity, duration, frequency, interest and length of intervention. Involvement of healthcare professionals including a multidisciplinary team, and a sense of enjoyment or motivation, related to competence, relatedness and autonomy also influence adherence [38, 39]. Apart from the direct effects of exercise and knowledge acquisition from the education sessions, the support and interaction with other survivors also help to improve mood and symptoms.

Despite its benefits, face-to-face supervision is resource-intensive and pose inconvenience, particularly for those suffering with symptoms during adjuvant treatment. Indeed, the most common reason for not attending at least half of exercise sessions in the centre-based group was feeling unwell or being hospitalized. A home-based programme is not without merit [40, 41] and measures can be taken to improve adherence, including attention to patient selection, exploration of patient characteristics, goals, evaluation of self-efficacy, social supports and other barriers and facilitators [13, 38]. Use of technology may ease the delivery of education sessions, augment appropriate prescription and allow for remote monitoring and supervision [38, 42].

In our study, the average cost of running the programme per participant was Singapore Dollar (SGD) 1,332.53 for the MDR group and SGD 149.90 for the HE group. While the cost may be considered acceptable, a hybrid model combining face-to-face sessions with telerehabilitation and telehealth monitoring may be explored to optimize the benefits of supervision, social and emotional support, with ease of access and resource management.

To our knowledge, this is the first study in Singapore and Asia to demonstrate the feasibility and efficacy of a supervised outpatient multi-dimensional rehabilitation and education programme during adjuvant cancer treatment. The strength of the study includes its prospective design with an active comparator, considered to be current best standard of care. Limitations of the study include the lack of randomization, the small sample size and the relatively high voluntary dropout rate in the HE group. We did not control for other medical comorbidities and the timing of intervention in relation to the cancer treatment for each participant, which may have contributed to the symptoms reported at a later stage, such as fatigue and nausea. Almost 2 in 3 breast cancer survivors who met criteria declined participation in this study. Future studies could target defining optimal dosage and intensity of exercise interventions, and exploring the effectiveness of telerehabilitation in exercise adherence monitoring and as an alternative mode of interaction with the healthcare professionals. Other methods to improve adherence to exercise during treatment could be explored, including providing programmes closer to the patients’ homes, engaging family support and increasing motivation for exercise through feedback and coaching by trainers or incorporating technology and gaming [43].

## Conclusion

Participation in a centre-based rehabilitation programme for breast cancer survivors undergoing adjuvant treatment is challenging. Nevertheless, the programme combining exercise and education was feasible and more effective in improving exercise endurance and earlier return to daily activities and participation for those with better attendance. Centre-based programme demonstrated better attendance and participant satisfaction.

## Data Availability

The datasets used and/or analysed during the current study are available from the corresponding author on reasonable request.

## Abbreviations

6MWT: 6-Minute walk test
BMI: Body mass index
CogPCA: Cognitive function- perceived cognitive abilities
CogPCI: Cognitive function- perceived cognitive impairment
CogOth: Cognitive function- comments from others
CogQOL: Cognitive function- impact of perceived cognitive impairments on quality of life
EORTC QLQ-C30: European organization for research and treatment of cancer quality of life questionnaire-core 30
FACT–Cog: Functional assessment of cancer therapy –cognitive function
FAI: Frenchay Activities Index
HE: Home-based exercise
iADL: Instrumental activities of daily living
LL: Lower limb
M6: 6-Months
MDR: Multidimensional rehabilitation programme
W0: Baseline
W12: 12-Weeks
RPE: Rate of perceived exertion
SGD: Singapore Dollar
UL: Upper limb
